# Visible Light Photoinitiator for 3D-Printing of Tough Methacrylate Resins

**DOI:** 10.3390/ma10121445

**Published:** 2017-12-19

**Authors:** Bernhard Steyrer, Philipp Neubauer, Robert Liska, Jürgen Stampfl

**Affiliations:** 1 Institute of Materials Science and Technology, TU Wien, 1060 Wien, Austria; bernhard.steyrer@tuwien.ac.at (B.S.); e1328884@student.tuwien.ac.at (P.N.); 2 Institute of Applied Synthetic Chemistry, TU Wien, 1060 Wien, Austria; robert.liska@tuwien.ac.at

**Keywords:** photopolymer, photoinitiator, additive manufacturing, digital light processing

## Abstract

Lithography-based additive manufacturing was introduced in the 1980s, and is still the method of choice for printing accurate plastic parts with high surface quality. Recent progress in this field has made tough photopolymer resins and cheap LED light engines available. This study presents the influence of photoinitiator selection and post-processing on the thermomechanical properties of various tough photopolymers. The influence of three photoinitiators (Ivocerin, BAPO, and TPO-L) on the double-bond conversion and mechanical properties was investigated by mid infrared spectroscopy, dynamic mechanical analysis and tensile tests. It was found that 1.18 wt % TPO-L would provide the best overall results in terms of double-bond conversion and mechanical properties. A correlation between double-bond conversion, yield strength, and glass transition temperature was found. Elongation at break remained high after post-curing at about 80–100%, and was not influenced by higher photoinitiator concentration. Finally, functional parts with 41 MPa tensile strength, 82% elongation at break, and 112 °C glass transition temperature were printed on a 405 nm DLP (digital light processing) printer.

## 1. Introduction

Lithography-based additive manufacturing (L-AM) was introduced in the 1980s, along with the commercialization of stereolithography. Shortly after, a few other processes were introduced for the additive manufacturing of polymeric parts. L-AM is still unmatched in terms of the resolution and surface quality of the final printed parts. L-AM relies on photopolymers, which are selectively cured by light in a layer-based process. Using conventional photopolymers for L-AM leads to either very rigid—but also brittle—parts; or soft parts, with a low glass transition temperature [1,2]. 4D-printing can make use of this low glass transition temperature in cross-linked photopolymers to enable shape-memory applications [3,4,5].

Recent research in this field has also focused on increasing the toughness of such photopolymerizable resins. One important approach is the reduction of crosslink density within the cured photopolymer [6]. This can be achieved in various ways, e.g., chain-transfer agents, monofunctional monomers, or not fully cured polymers. Incomplete curing would lead to a change in properties over time [7], and the insufficient long-term stability of the printed parts [8]. Common chain-transfer agents are either based on thiol-ene systems, which have a bad odor and poor storage stability, or custom-synthesized molecules, which are not commercially available. These systems are therefore not yet used in commercial resins. Tough photopolymer resins based on monofunctional monomers are already available. The Cubiflow resin used for this study is based on such monofunctional monomers.

Resins for L-AM are mostly based on (meth)acrylates, which are cured by a free radical photoinitiator. Once exposed to the appropriate light source, these photoinitiators create radicals and start the polymerization of the (meth)acrylates. Recent progress in LED light-source technology has led to a variety of photoinitiators for LED-curing applications [9,10,11]. This has made economical 405 nm LED light sources available for L-AM [12]. 

Using these LED-based light sources for L-AM with tough resins could lead to a competitive manufacturing system. A resin for this system requires an optimized photoinitiator and light absorber to provide accurate curing [13]. Conventional dimethacrylates are able to reach their gel point below 20% conversion [14]. This would be sufficient curing for the printing process, but would lead to inferior material properties. Therefore, it is important to provide high conversions for such photopolymers by post-curing after printing. 

Additional light exposure and heating up the photopolymer are the two common options for post-curing. Heating to above the glass transition temperature will lead to bad dimensional stability, and should be avoided if possible [15]. Thermal post-curing also leads to higher shrinkage strains than UV-light curing, which provides a more uniform shrinkage [16]. Higher conversions usually lead to an increase in modulus and tensile strength, while decreasing impact strength and elongation at break [17]. It is therefore necessary to determine the properties in the fully cured state.

There are several methods for analyzing the curing state of 3D-printed photopolymers, including differential scanning calorimetry (DSC), spectroscopic methods, and mechanical analysis [18,19,20,21,22,23]. While spectroscopic methods give direct chemical information about the double-bond conversion, there are several limitations for measuring printed parts. For example, particle-filled resins prevent light transmission, and volatile compounds would interfere with surface measurements. On the other hand, mechanical testing would often represent the whole cross-section, and fillers do not interfere with the measurement. Mechanical analysis will only give relative information about the curing state, and cannot directly confirm full curing of a photopolymer. Combining mechanical and chemical analysis gives the best interpretable results for 3D-printed photopolymers [17]. 

The aim of this study is to select a suitable photoinitiator and concentration range for printing a monofunctionally based photopolymer with a 405 nm light engine. Finally, the application of the findings will be demonstrated by printing three-dimensional objects.

## 2. Results

### 2.1. Spectroscopic Analysis

**Figure 1:**
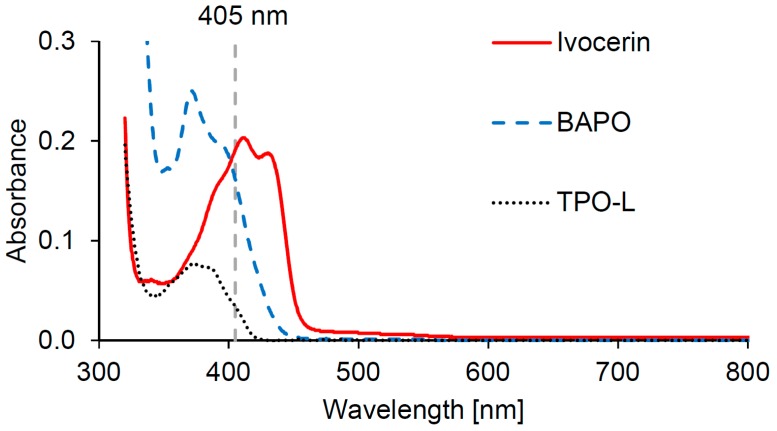
Absorbance spectra of 0.01 wt % photoinitiators in IBOMA.

The UV-Vis spectra of the three used photoinitiators Ivocerin, BAPO and TPO-L were measured in isobornyl methacrylate (IBOMA), as this would best represent the absorbance in the Cubiflow resin. The spectra show that all three photoinitiators absorb in the desired range of around 405 nm (Figure 1). TPO-L shows rather low absorption at 405 nm compared to Ivocerin and BAPO.

### 2.2. Photocuring Studies

**Figure 2:**
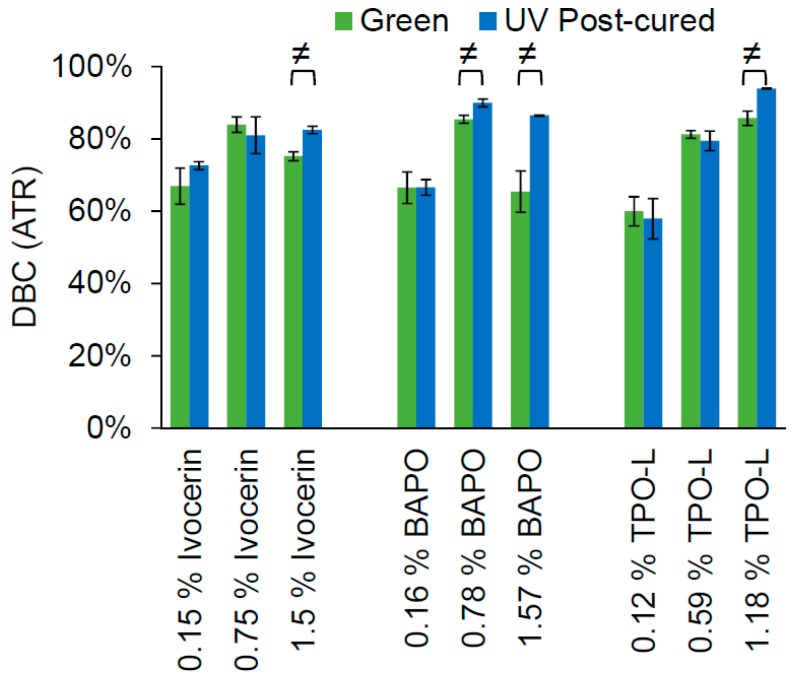
Double-bond conversion (DBC) of the cured specimens measured by ATR in MIR. Marks (≠) show significant difference (*t*-test, α = 0.05) between green and post-cured values.

The photoinitiators were used in three equimolar concentrations to cure 2 mm-thick samples of the Cubiflow resin. A 400 nm LED-array was used to simulate the printing process (green), and a mercury lamp UV-curing oven was used for post-curing the samples (UV post-cured). The double-bond conversion was measured in mid infrared (MIR) with an ATR (attenuated total reflection) set-up on the non-exposed side of the samples (Figure 2). This would represent the curing state at 2 mm depth and exclude contact to oxygen and its inhibition during the curing process. Post-curing showed a statistically significant increase in double-bond conversion (*t*-test, α = 0.05) for the highest concentration of each photoinitiator, and for the middle concentration of BAPO.

The lowest concentration of each photoinitiator would not be enough to reach a high level of double-bond conversion. Even UV post-curing does not show a significant increase (*t*-test, α = 0.05) in double-bond conversion. The highest concentration of each photoinitiator leads to significant conversion increase after post-curing. This means that there are uncured photoinitiator molecules left, which would be triggered by the higher light intensity during post-curing. It is noticeable that the green conversion with Ivocerin and BAPO is higher at the medium concentration than at the highest concentration. This shows that the higher absorption at 400 nm of these two photoinitiators would prevent the light from fully penetrating the sample, which could also affect mechanical properties. This does not occur with TPO-L, which has the lowest absorption of these three photoinitators, at 400 nm.

### 2.3. Tensile Test

**Figure 3:**
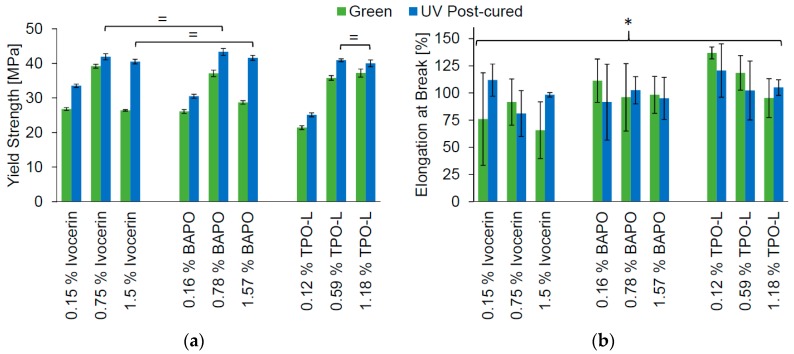
Influence of photoinitiator and its concentration on yield strength (**a**) and elongation at break (**b**) from tensile tests conducted in accordance with ISO 527-1:2012. Marks (=) show values of no significant difference for post-cured groups; * ANOVA showed no significant difference between all post-cured groups (*p* = 0.09).

The tensile test of the Cubiflow photopolymer was done according to ISO 527-1, with type 5B specimens. The samples were tested as cast (green), and after UV post-curing. The yield strength showed significant difference (α = 0.05) between almost all groups (Figure 3a). Even the groups with the highest photoinitiator concentration and post-curing showed a significant difference (analysis of variance—ANOVA, *p* = 0.01). Only the highest concentrations with post-curing of Ivocerin and TPO-L did not show a significant difference (*t*-test, *p* = 0.27). There was no significant difference (ANOVA, *p* = 0.09) between all groups for elongation at break (Figure 3b).

The yield strength decreased for green specimens with higher concentrations of Ivocerin and BAPO, just as was seen with double-bond conversion in Figure 2. This shows that it was not only the underneath surface, but the whole cross-section, that was not fully cured at high concentrations of Ivocerin and BAPO. Hence, TPO-L would give a higher green strength when higher concentrations of photoinitiator were used. The highest concentration of TPO-L would be the first choice for printing, since this also leads to the highest conversion after post-curing.

**Figure 4:**
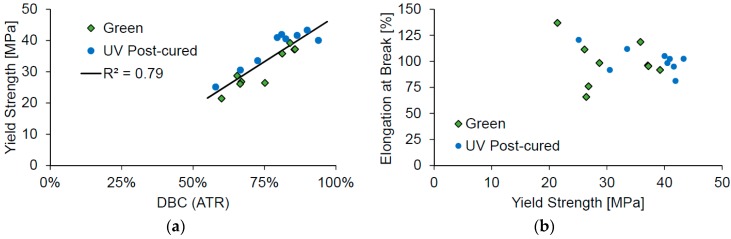
Comparing the measured yield strength to the corresponding double-bond conversion (**a**); and comparing the elongation at break to the yield strength (**b**).

Comparing the results from the tensile tests to the double-bond conversion shows that a higher conversion would also result in a higher yield strength (Figure 4a). On the other hand, the elongation at break does not show any correlation to the measured yield strength (Figure 4b).

### 2.4. Dynamic Mechanical Analysis

**Figure 5:**
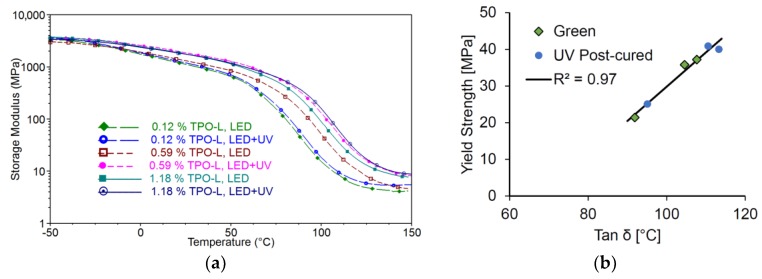
Storage modulus measured by DMA (**a**); and the measured glass transition temperature (tan δ) compared to the yield strength (**b**).

The thermomechanical properties and the glass transition temperature of the Cubiflow resin with TPO-L as photoinitiator were measured by Dynamic Mechanical Analysis (DMA). The storage modulus and the glass transition temperature increase with higher photoinitiator concentration and post-curing (Figure 5a). This also correlates with the tensile properties (Figure 5b).

This shows that both glass transition temperature and yield strength are good relative indicators for the curing condition within the photopolymer.

### 2.5. 3D Printing

**Figure 6:**
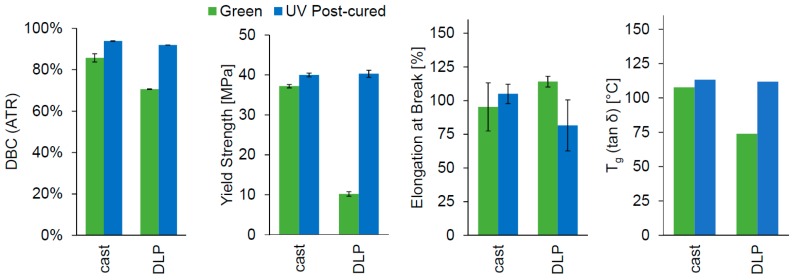
Comparison of DLP-printed photopolymer samples to cast photopolymer samples (1.18 wt % TPO-L). Each was tested as printed/cast (green) and after UV post-curing.

A lithographic printer set-up based on a 405 nm DLP light engine was used for printing the Cubiflow resin with 1.18 wt % TPO-L. The same specimen geometries were used for printing as for the cast specimens (ISO 527 type 5B and 30 × 4 × 2 mm³). After post-curing, the printed parts showed similar properties to the cast specimens (Figure 6). The yield strength (*t*-test *p* = 0.47) and elongation at break (*t*-test *p* = 0.09) did not show significant difference between cast and printed samples after post-curing. The results for double-bond conversion (94% and 92%) and glass transition temperature (113 °C and 112 °C) were also very similar. However, the green parts showed significant difference for all tested values, except elongation at break.

The conversion of DLP printed parts was also verified by extraction tests. These showed a gel content of 81.9 ± 0.5% in green parts and 97.9 ± 0.4% in post-cured parts.

**Figure 7:**
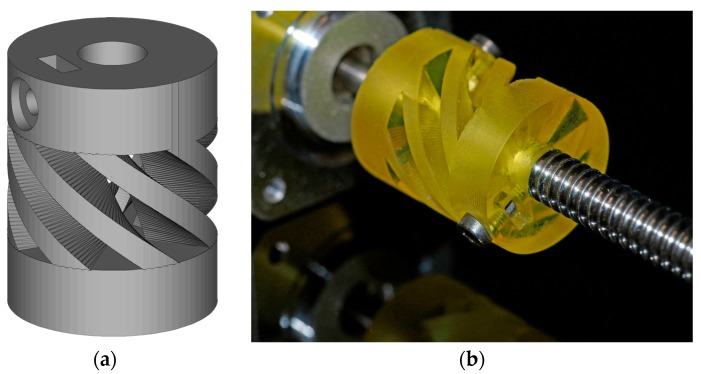
CAD model of a functional shaft coupling (**a**), and the final assembled 3D-printed part (**b**). Printed with 1.18 wt % TPO-L.

Finally, the Cubiflow resin with 1.18 wt % TPO-L was used to produce a functional shaft coupling (Figure 7).

The final 3D-printed part was precise enough to be used without any mechanical post-treatment. Even the tessellation triangles from the CAD model are noticeable on sloped surfaces. This shows that the Cubiflow resin in combination with the right amount of TPO-L can be used to print very accurate parts.

## 3. Discussion

It was demonstrated in this study that TPO-L was well suited to printing accurate parts with full mechanical strength on a 405 nm DLP printer. A high concentration of photoinitiator is necessary in order to reach high rates of double-bond conversion. Ivocerin and BAPO have higher absorption at 405 nm than TPO-L, and therefore reduce light penetration at this wavelength. This reduces green conversion within the photopolymer for higher concentrations of these two photoinitiators. In addition to lower conversion, less light penetration may also have an additional effect on curing kinetics and chain length. A more sensitive approach (e.g., photorheometer studies) would be necessary to analyze this effect in future work. In this study, the best results were achieved with 1.18 wt % of TPO-L and UV post-curing. 

**Figure 8:**
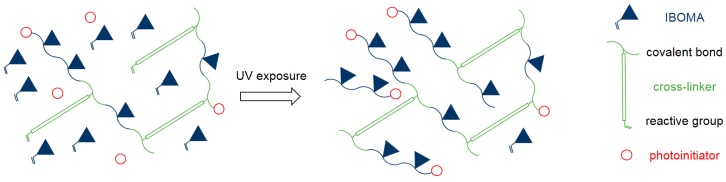
Uncured IBOMA possibly acting as plasticizer in the green part (**left**), and further curing leading to more covalent-bond IBOMA molecules (**right**).

Results from tensile testing confirmed these findings. After post-curing, a higher photoinitiator concentration led to higher double-bond conversion and higher yield strength, while elongation at break was not affected. Further curing usually causes increased tensile strength and decreased elongation at break for conventional photopolymers [7,17]. Conventional resins mostly consist of di- or multifunctional monomers where further curing causes more cross-links. The high content of monofunctional IBOMA in the Cubiflow resin can act as plasticizer in its uncured state. Reaching higher conversions of IBOMA reduces this plasticizing effect, while increasing the molecular weight of the photopolymer without directly adding additional cross-links (Figure 8). This explains why higher photoinitiator concentration has such a great effect on yield strength, while not reducing the elongation at break. 

Thermomechanical analysis and tensile testing showed high correlation for glass transition temperature and yield strength. This shows that both values could be used as relative indicators for the curing state within the photopolymer.

The printed green parts show significant difference from the cast green parts. This is caused by the differing specimen preparation. For printing, 50 µm-thick layers were consecutively exposed for 40 s, just enough to cure thin layers for a precise printing process. The cast specimens were exposed in bulk for 300 s to analyze how the photoinitiators would perform at this particular wavelength. The actual testing should therefore be done with post-cured specimens, since all printed parts would be post-cured before their application anyway. 

The post-cured results from cast and DLP printed specimens showed no significant difference for yield strength and elongation at break. The glass transition temperature was very similar (113 °C and 112 °C), and could be considered identical. The difference in double-bond conversion (94% and 92%) is statistically significant because the deviation is unusual small (±0.1% and ±0.02%). This is not representative for the accuracy of the ATR measurement considering the deviation of other results (up to ±5.1%). Therefore, cast and printed specimens have shown equivalent results after post-curing.

Extraction tests (97.9 ± 0.4% gel content) confirmed the high conversion of the post-cured parts.

Future work should focus on accelerating the printing process to reduce the production time. This could be done either by increasing light intensity or tuning the light absorber.

Finally, an accurate printing process was demonstrated with a 405 nm DLP printer. Functional parts were printed with 1.18 wt % TPO-L.

## 4. Materials and Methods

### 4.1. Materials

**Figure 9:**
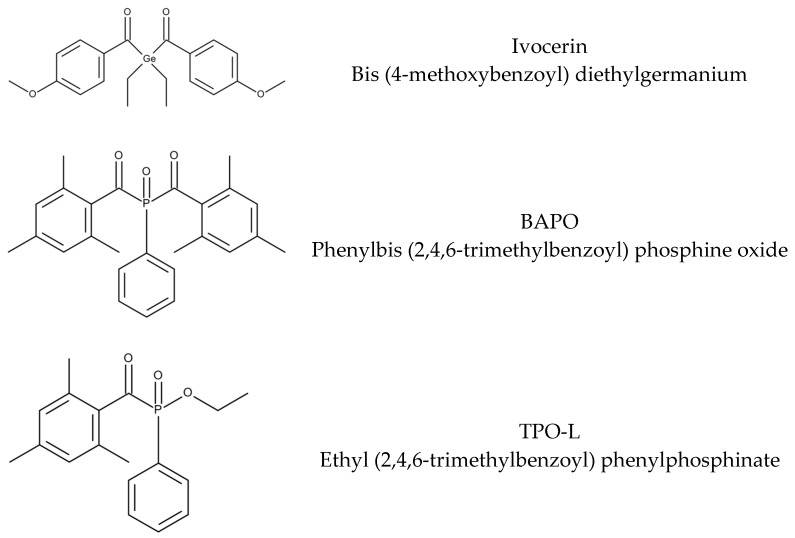
Photoinitiators used in this study.

The materials for this study were used as received: Ivocerin (Ivoclar Vivadent, Schaan, Liechtenstein), Omnirad 819 and Omnirad TPO-L (IGM-Resins, Walwijk, The Neatherlands), Isobornyl methacrylate (IBOMA, Sigma-Aldrich, Vienna, Austria), and UV-1995 (Eutech Chemicals, Taipei, Taiwan). The chemical structures of the photoinitiators are shown in Figure 9. The Cubiflow photopolymer was provided by Cubicure without photoinitiator. Cubiflow is a commercial available 3D-printing resin based on IBOMA and difunctional methacrylates.

All work and handling until post-curing was carried out in a yellow-light laboratory to avoid blue/UV light exposure.

### 4.2. Preparation and Curing of Specimens

The photoinitiators were used in three equimolar concentrations:

- Ivocerin: 0.15 wt %, 0.75 wt %, and 1.5 wt %
- BAPO: 0.16 wt %, 0.78 wt %, and 1.57 wt %
- TPO-L: 0.12 wt %, 0.59 wt %, and 1.18 wt %.

Each resin was prepared with 10 g Cubiflow, and stirred until the photoinitiators were fully dissolved. A casting mould for DMA test bars (2 × 4 × 30 mm^3^) and tensile test bars (ISO 527-2:1996 type 5B) was prepared from white non-transparent Wacker Elastosil 4503 (München, Germany). For DLP-like curing conditions, a 395–400 nm LED array (ADJ UV Flood 36) was used. The solutions were cast and cured for 300 s at 10 mW cm^−2^. For additional post-curing, a UV flood curing system (Uvitron Intelliray 600, West Springfield, MA, USA) was used. A set of specimens from each composition was post-cured for 300 s at 100% intensity. Each specimen was only exposed from one side, the underside did not receive any direct light exposure.

### 4.3. Spectroscopic Measurements

UV/VIS-spectra of the photoinitiators were measured with a UV/VIS-spectrometer (Shimadzu UV/VIS 1800, Kyoto, Japan) as 0.01 wt % solution in IBOMA.

The double-bond conversion (DBC) was measured by ATR with a FTIR-spectrometer (Perkin Elmer Spectrum 65, Waltham, MA, USA). The uncured resins were measured as reference before exposure. A DMA bar from each resin was measured both after LED-curing and after post-curing. The underside of the bars was measured at three different positions. The peak areas for C=C double-bonds at 1637 cm^−1^ (Baseline 1660–1621 cm^−1^) and C=O at 1714 cm^−1^ (Baseline 1819–1621 cm^−1^) were analyzed. The double-bond conversion was calculated by:

(1) $$\mathrm{DBC}\;=\;1-\frac{\frac{Cured_{1637}}{Cured_{1714}}}{\frac{Resin_{1637}}{Resin_{1714}}}$$

Mean value and standard deviation were calculated from the three measured spots.

### 4.4. Gel Content

The gel content of the printed plates was analyzed by extraction with methyl t-butylether (MTBE). Three plates of the green and post-cured material were used. The plates were extracted with excessive MTBE at 23 °C for 24 h. The plates were removed from the MTBE and stored at 23 °C for 72 h to prevent cracking. The remaining MTBE was removed at 85 °C until constant weight was reached (~7 days). The gel content was calculated by:

(2) $$\mathrm{Gel}\;\mathrm{content}\;=\;\frac{m_{\left(after\;extraction\right)}}{m_{\left(before\;extraction\right)}}$$

Mean value and standard deviation were calculated from three plates.

### 4.5. Mechanical Properties

**Figure 10:**
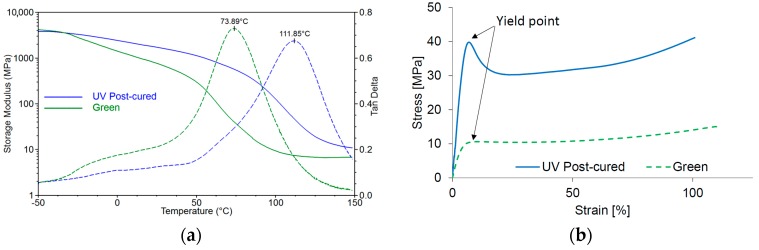
Testing of DLP-printed samples (1.18 wt % TPO-L): (**a**) Storage modulus (**solid line**) and the tan δ-curve (**dashed line**) for green and post-cured samples. The peak of the tan δ-curve was analyzed; (**b**) Stress-strain curves from tensile tests. The samples show a yield point, which was used for the analysis.

The DMA specimens were analyzed with a Dynamic Mechanical Analyzer (TA Instruments 2980) from −50 to 150 °C with a heating rate of 3 °C min^−1^. The test was done with a frequency of 1 Hz, an amplitude of 20 µm and a preload force of 0.1 N. The peak of the tan δ curve was used as an estimation of the glass transition temperature (Figure 10).

Five tensile specimens of each cured photopolymer were tested on a Zwick Z050 (Zwick Roell, Ulm, Germany) according to ISO 527-1:2012 with 5 mm min^−1^. The photopolymers showed a yield point as seen in Figure 10.

Mean value and standard deviation were calculated for the yield strength and elongation at break from five specimens for each photopolymer.

### 4.6. 3D Printing

The photopolymer resin was printed with a self-made setup based on digital light processing (DLP) with 405 nm and diamond-WXGA resolution. For printing, 50 g of Cubiflow with 1.18 wt % TPO-L as photoinitiator and 0.05 wt % UV-1995 as UV-Vis absorber were prepared. Curing was done with an intensity of 4 mW cm^−2^ and 40 s exposure per layer. The layer height was set to 50 µm. The parts were cleaned with pressurized air and paper towels.

**Figure 11:**
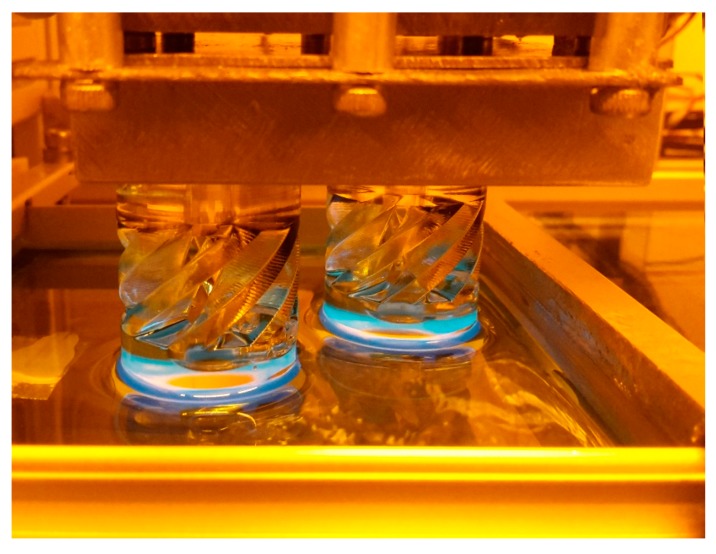
Printing of two shaft couplings with the 405 nm DLP set-up. Moving platform is on top and a transparent vat containing the resin on the bottom.

Two sets of tensile test bars (ISO 527-2:1996 type 5B) and DMA test bars (2 × 4 × 30 mm^3^) were printed. The specimens were oriented flat on the XY-plane and printed without support structures. One set was post-cured for 300 s in the UV flood curing system, and the other set was used as printed (green). To demonstrate the accuracy of the printing process a functional shaft coupling [24] was printed (Figure 11).

The shaft couplings were cleaned with pressurized air and paper towels. Due to the higher wall thickness, the parts were post-cured from 4 different angles. This was done in a UV flood curing system for 300 s on each side.

## 5. Conclusions

**Figure 12:**
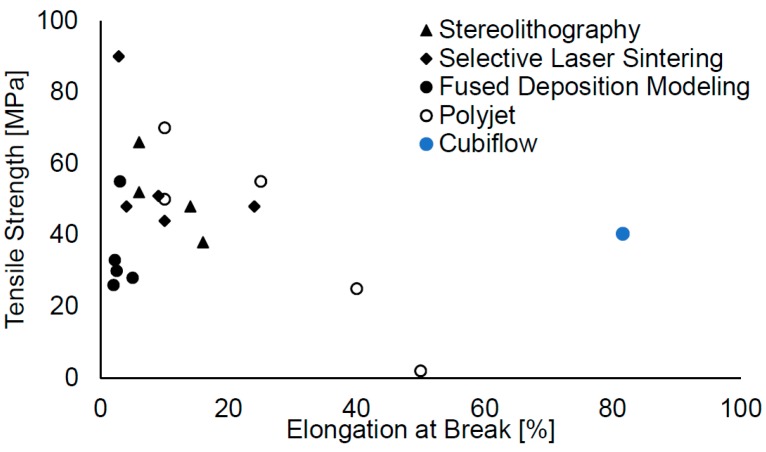
Comparison of the Cubiflow tensile strength and elongation at break results with values from other additive manufacturing polymer materials [2].

The material used in this study were able to be printed with low-cost 405 nm DLP light engines, while providing good mechanical properties for the printed parts. A tensile strength of 41 MPa, elongation at break of 81% and a glass transition temperature of 112 °C are very competitive. Figure 12 provides a comparison to recent literature values for other additive manufacturing polymers [2].

Processing such materials with low-cost DLP printers enables new possibilities for additive manufacturing. For example, production of individual parts or small batch sizes is finally at eye level with injection molding in terms of surface quality and mechanical properties.
